# Incidence and sociodemographic characteristics of eczema diagnosis in children: A cohort study

**DOI:** 10.1016/j.jaci.2017.12.997

**Published:** 2018-05

**Authors:** Lu Ban, Sinéad M. Langan, Katrina Abuabara, Kim S. Thomas, Alyshah Abdul Sultan, Tracey Sach, Emma McManus, Miriam Santer, Sonia Ratib

**Affiliations:** a Centre of Evidence Based Dermatology, University of Nottingham, Nottingham, United Kingdom; b NIHR Nottingham Biomedical Research Centre (BRC), Nottingham University Hospitals NHS Trust and the University of Nottingham, Nottingham, United Kingdom; c Department of Non-communicable Disease Epidemiology, London School of Hygiene and Tropical Medicine, London, United Kingdom; d Department of Dermatology, University of California, San Francisco (UCSF), San Francisco, Calif; e Arthritis Research UK Primary Care Centre, Research Institute for Primary Care & Health Sciences, Keele University, Staffordshire, United Kingdom; f Health Economics Group, University of East Anglia, Norwich, United Kingdom; g Primary Care & Population Sciences, University of Southampton, Southampton, United Kingdom

To the Editor:

We report the results of a large population-based cohort study examining the incidence of clinically diagnosed eczema in children and the variations by sociodemographic characteristics. Eczema (also known as atopic eczema/dermatitis^1^) affects up to 1 in 5 children^2^ and is associated with high morbidity.^3^

There are limited estimates on the incidence of eczema and how the incidence varies by sociodemographic factors, which is important for generating hypotheses regarding the disease etiology and for health service planning. To address this issue, we examined the incidence of eczema diagnosis in children aged 0 to 17 years between April 1, 1997, and March 31, 2015, using the Clinical Practice Research Datalink (CPRD).^4^ CPRD is a routinely collected primary care database in the United Kingdom covering approximately 7% of the UK population.^4^ CPRD has been linked to the Hospital Episode Statistics, a secondary health care administrative database in England, and is broadly representative of the general UK population regarding age, sex, and lifestyle-related factors.^4^, ^5^

We defined a child as having eczema if he or she had 1 diagnostic code for eczema with at least 2 eczema-related treatment codes on separate days within 3 months before or 1 year after the eczema diagnosis (for additional details, see this article's Online Repository at www.jacionline.org). The earliest date of an eczema diagnosis was defined as the incidence date. Previous research^6^ has shown that the combination of 1 eczema diagnostic code with 2 eczema-related treatment codes on separate days at any time gives a 90% (95% CI, 83%-96%) positive predictive value for identifying prevalent eczema in children. We excluded children registered with their current primary care practice after 3 months of birth or children with a history of eczema before the start of the study to minimize the risk of misclassifying recurrent eczema events as first events (for additional details, see Fig E1 in this article's Online Repository at www.jacionline.org).

We calculated incidence rates per 100 person-years and adjusted rate ratios (aRRs) for age (<1 year old, 1-4 years old, 5-17 years old), sex, socioeconomic status (defined as quintiles of the patient-level English Index of Multiple Deprivation^7^), and ethnicity (when available) using Poisson regression modeling with mutual adjustment (see Table I). Because people with different sociodemographic characteristics could have different health-seeking behaviors, we also adjusted for the number of annual consultations in the study follow-up period to minimize potential ascertainment bias. We also examined the incidence rate and aRR for calendar year adjusted for age, sex, and socioeconomic status. We examined whether there was evidence of statistical interaction between age and sex, socioeconomic status, and ethnicity using the likelihood ratio test (*P* < .05). Because the quality of ethnicity recording in the Hospital Episode Statistics–linked CPRD population is only comparable to the UK population for people registered after 2006,^8^ for any analysis using ethnicity data we excluded children registered before April 1, 2006, and conducted a complete case analysis. To test the robustness of our results, we conducted 4 sensitivity analyses (see this article's Online Repository at www.jacionline.org). The study protocol was approved by the Independent Scientific Advisory Committee (Protocol No: 16_056) and published here: https://www.cprd.com/isac/Protocol_16_056.asp.

**Table I:** Incidence rates and rate ratios of eczema by different sociodemographic factors stratified by age (N = 675,087)

Sociodemographic factor	<1-y-olds (n of eczema = 55,525)		1-4-y-olds (n of eczema = 34,729)		5-17-y-olds (n of eczema = 7,828)	
	Rate∗ (95% CI)	aRR† (95% CI)	Rate∗ (95% CI)	aRR† (95% CI)	Rate∗ (95% CI)	aRR† (95% CI)
Sex						
Male	15.9 (15.7-16.1)	1.3 (1.3-1.4)‡	2.9 (2.9-3.0)	0.9 (0.9-1.0)	0.4 (0.3-0.4)	0.8 (0.7-0.8)‡
Female	11.7 (11.5-11.8)	Reference	3.0 (2.9-3.0)	Reference	0.5 (0.5-0.5)	Reference
Index of Multiple Deprivation						
1 (least deprived)	15.5 (15.3-15.8)	1.2 (1.2-1.3)‡	3.2 (3.2-3.3)	1.2 (1.1-1.3)‡	0.4 (0.4-0.4)	1.0 (0.9-1.1)
2	13.7 (13.5-14.0)	1.1 (1.0-1.1)	3.0 (2.9-3.1)	1.1 (1.0-1.1)	0.4 (0.4-0.4)	1.0 (0.9-1.1)
3	13.5 (13.3-13.8)	1.0 (1.0-1.1)	2.9 (2.8-3.0)	1.0 (0.9-1.1)	0.4 (0.4-0.4)	1.0 (0.9-1.1)
4	13.1 (12.8-13.3)	1.0 (1.0-1.1)	2.8 (2.8-2.9)	1.0 (0.9-1.1)	0.4 (0.4-0.5)	1.0 (1.0-1.1)
5 (most deprived)	12.9 (12.6-13.2)	Reference	2.8 (2.7-2.9)	Reference	0.4 (0.4-0.5)	Reference
Ethnicity§	n of eczema = 25,593		n of eczema = 12,862		n of eczema = 391	
White	12.4 (12.2-12.6)	Reference	3.3 (3.2-3.3)	Reference	0.5 (0.4-0.5)	Reference
Black Caribbean	28.8 (25.6-32.4)	2.5 (2.3-2.9)‡	5.4 (4.5-6.6)	2.0 (1.6-2.4)‡	1.5 (0.6-4.0)	3.5 (1.3-9.3)‡
Bangladeshi	30.4 (27.2-34.1)	2.5 (2.3-2.8)‡	5.3 (4.3-6.5)	1.4 (1.1-1.7)‡	1.0 (0.3-3.0)	1.6 (0.5-5.1)
Chinese	41.7 (36.9-47.2)	3.4 (3.0-3.8)‡	4.6 (3.4-6.2)	1.6 (1.2-2.2)‡	0.7 (0.1-5.1)	1.9 (0.3-13.3)
All other ethnic groups combined‖	20.8 (20.2-21.4)	1.7 (1.6-1.8)‡	3.9 (3.8-4.1)	1.1 (1.0-1.2)	1.0 (0.8-1.2)	1.9 (1.5-2.5)‡

∗ Rate per 100 person-years.

† For sex, model adjusted for Index of Multiple Deprivation and the number of annual consultations during the study follow-up period; for Index of Multiple Deprivation, model adjusted for sex and the number of annual consultations during the study follow-up period; for ethnicity (available only for children registered after 2006), model adjusted for sex, Index of Multiple Deprivation, and the number of annual consultations during the study follow-up period.

‡ *P* < .05.

§ Restricted to children with current registration dates on or after April 1, 2006 (N = 303,327 of which 48,301 with eczema), and a complete case analysis was conducted first by excluding 55,529 (18.3%) children with missing ethnicity data (N = 247,798).

‖ Including mixed, black African, black other, Indian, Pakistani, Asian other, and other children (eg, Egyptian).

The study population consisted of 675,087 children of which 98,082 (14.5%) had a first clinical diagnosis of eczema. Compared with children without eczema, children with eczema had a slightly longer follow-up period and a higher annual consultation rate (see Table E1 in this article's Online Repository at www.jacionline.org).

The incidence rate by calendar year remained stable in the period 1997 to 2015 (see Fig E2 in this article's Online Repository at www.jacionline.org) and the aRR for each additional calendar year was 1.0 (95% CI, 1.0-1.0). The incidence rate of eczema was highest in the first year of life (13.8 per 100 person-years; 95% CI, 13.7-13.9) and decreased substantially afterward (Fig 1). We found statistically significant interaction between age and other sociodemographic factors (*P* < .001). There was a 30% higher incidence rate in boys than in girls in children younger than 1 year (aRR, 1.3; 95% CI, 1.3-1.4) and a 20% lower rate in boys than in girls for children 5 years or older (aRR, 0.8; 95% CI, 0.7-0.8) (Table I). On comparing the incidence rate in children of the lowest socioeconomic status with that in children of the highest socioeconomic status, we found that the latter had a 20% higher incidence rate in the younger age groups (aRR, 1.2; 95% CI, 1.2-1.3 in <1-year-olds; aRR, 1.2; 95% CI, 1.1-1.3 in 1-4-year-olds); such difference however was not observed in children 5 years or older (Table I). Moreover, the incidence of clinically diagnosed eczema in the first year of life was 2- to 3-fold higher in Chinese children (aRR, 3.4; 95% CI, 3.0-3.8), Bangladeshi children (aRR, 2.5; 95% CI, 2.3-2.8), and Black Caribbean children (aRR, 2.5; 95% CI, 2.3-2.9) compared with white children (Table I). The incidence decreased by age for all ethnic groups but generally remained higher in nonwhite children than in white children (see Table E2 in this article's Online Repository at www.jacionline.org). Results from the sensitivity analyses were all similar compared with the main analysis (see Table E3, Table E4, Table E5 in this article's Online Repository at www.jacionline.org).

**Fig 1:**
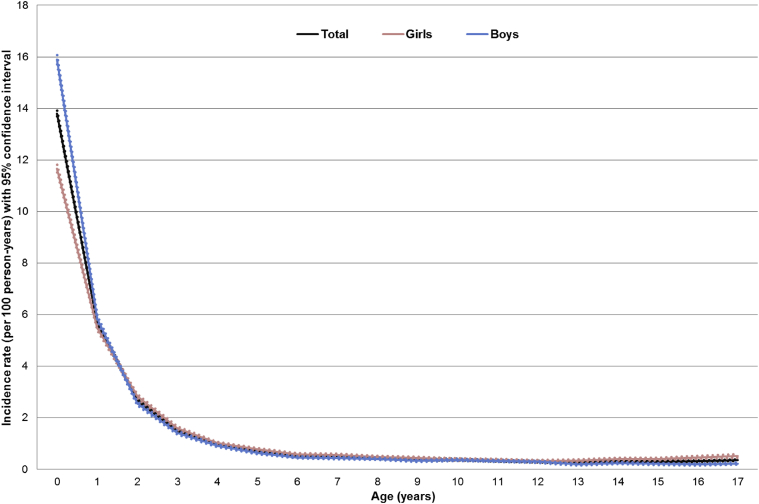
Incidence rate (per 100 person-years) of eczema by age and sex, N = 675,087 (dotted lines showing 95% CI).

Our study shows that the incidence of eczema varies substantially by age and is highest in the first year of life, especially in boys, Chinese, Bangladeshi, and Black Caribbean children, and children of high socioeconomic status. The study confirms the previously reported link between high socioeconomic status and the occurrence of eczema,^9^ and also reports novel findings on ethnic group and sex differences. The former could be due to different environmental risk factors such as diet, living conditions at home, or decreased exposure to ultraviolet light.E1, E2, E3 The latter may be potentially due to different immune responses of boys and girls in early childhood,^E4^ but different environment exposures such as differing exposures to soap/shampoo products^E5^ at older age.

The main strength of our study is the large sample size, which has allowed us to examine interactions with age. A potential limitation is ascertainment bias, but we have tried to minimize this by adjusting for the number of annual consultations during the study follow-up period in all the analyses.

In conclusion, our findings highlight the early onset of eczema in children, with higher incidence found in boys, Chinese, Bangladeshi, and Black Caribbean children, and those with high socioeconomic status. With new prevention approaches potentially available^E6^ and early intervention trials currently underway,^E7^ our study may help policymakers identify high-risk children and better allocate limited health care resources.

## Figures and Tables

**Fig E1:**
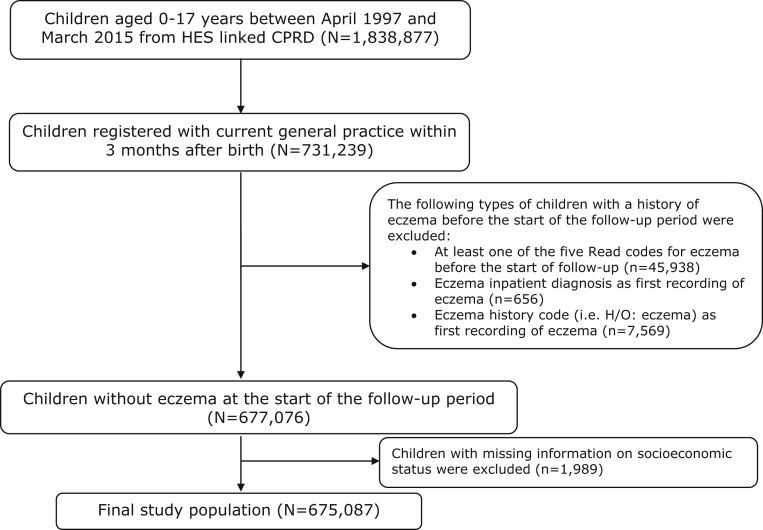
Constructing the final study population (N = 675,087). *H/O*, History of; *HES*, Hospital Episode Statistics.

**Fig E2:**
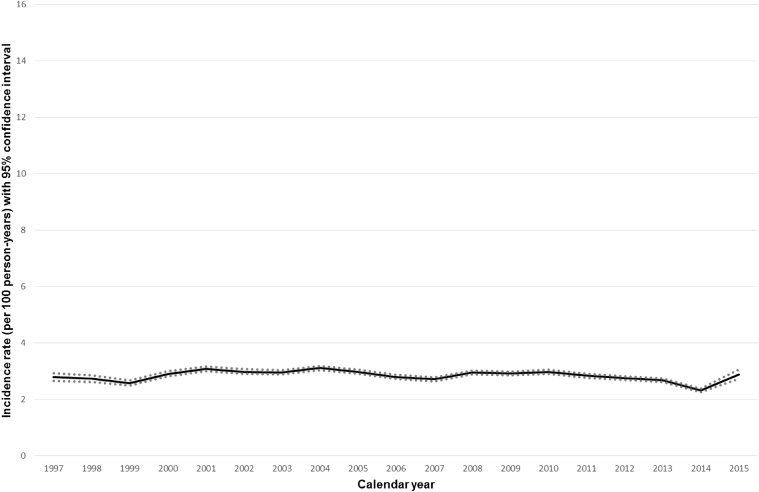
Incidence rate (per 100 person-years) of eczema by calendar year, N = 675,087.

**Table E1:** Characteristic of children without and with eczema (N = 675,087)

Characteristic	Children without eczema (n = 577,005)	Children with eczema (n = 98,082)
Age at registration with current GP (mo), median (IQR)	0.77 (0.20-1.40)	0.80 (0.37-1.33)
Age at start of prospective follow-up (mo), median (IQR)	1.30 (0.63-16.67)	0.90 (0.47-1.57)
Average length of prospective follow-up (mo), median (IQR)	54.73 (20.22-112.00)	74.27 (37.53-125.93)
Sex, male	293,828 (50.92)	53,124 (54.16)∗
Index of Multiple Deprivation		
1 (least deprived)	123,060 (21.33)	24,334 (24.81)∗
2	120,913 (20.96)	20,646 (21.05)
3	110,121 (19.08)	18,019 (18.37)
4	117,033 (20.28)	18,442 (18.80)
5 (most deprived)	105,878 (18.35)	16,641 (16.97)
Ethnicity†	n = 255,026	n = 48,301
White	176,964 (84.69)	30,761 (79.19)∗
Mixed	7,824 (3.74)	1,604 (4.13)
Black Caribbean	1,094 (0.52)	378 (0.97)
Black African	4,779 (2.29)	1,172 (3.02)
Black other	1,030 (0.49)	323 (0.83)
Indian	4,560 (2.18)	1,319 (3.40)
Bangladeshi	1,084 (0.52)	396 (1.02)
Pakistani	4,265 (2.04)	1,114 (2.87)
Chinese	704 (0.34)	294 (0.76)
Asian other	2,961 (1.42)	790 (2.03)
Other	3,687 (1.76)	695 (1.79)
Missing	46,074	9,455
Annual consultation rate‡, median (IQR)	3.50 (1.84-6.36)	10.29 (5.92-16.23)∗

Values are n (%) unless otherwise indicated.

*GP*, General practitioner; *IQR*, interquartile range.

∗ *P* < .05.

† Restricted to children with current registration dates on or after April 1, 2006 (N = 303,327 of which 48,301 with eczema), and a complete case analysis was conducted first by excluding 55,529 (18.3%) children with missing ethnicity (N = 247,798).

‡ Number of consultations per year during the study follow-up period.

**Table E2:** Incidence rates and rate ratios of eczema by ethnicity stratified by age (N = 247,798 with complete data on ethnicity)

Ethnicity	<1-y-olds (n of eczema = 25,593)		1-4-y-olds (n of eczema = 12,862)		5-17-y-olds (n of eczema = 391)	
	Rate∗ (95% CI)	aRR† (95% CI)	Rate∗ (95% CI)	aRR† (95% CI)	Rate∗ (95% CI)	aRR† (95% CI)
White	12.4 (12.2-12.6)	Reference	3.3 (3.2-3.3)	Reference	0.5 (0.4-0.5)	Reference
Mixed	17.7 (16.7-18.8)	1.5 (1.4-1.6)	3.7 (3.3-4.0)	1.1 (1.0-1.3)	0.6 (0.3-1.0)	1.2 (0.7-2.3)
Black Caribbean	28.8 (25.6-32.4)	2.6 (2.3-2.9)‡	5.4 (4.5-6.6)	2.0 (1.6-2.4)	1.5 (0.6-4.0)	3.5 (1.3-9.4)‡
Black African	22.3 (20.8-23.8)	2.0 (1.8-2.1)	3.9 (3.5-4.4)	1.3 (1.2-1.5)	1.1 (0.6-1.9)	2.6 (1.5-4.5)
Black other	29.6 (26.1-33.5)	2.6 (2.3-3.0)	4.0 (3.1-5.1)	1.4 (1.1-1.8)	2.4 (1.2-4.8)	5.8 (2.8-11.8)
Indian	24.3 (22.9-25.9)	1.8 (1.6-2.0)	4.2 (3.7-4.6)	1.2 (1.1-1.4)	0.9 (0.6-1.6)	1.8 (1.1-3.1)
Bangladeshi	30.4 (27.2-34.1)	2.5 (2.3-2.8)	5.3 (4.3-6.5)	1.4 (1.1-1.7)	1.0 (0.3-3.0)	1.6 (0.5-5.1)
Pakistani	20.6 (19.2-22.1)	1.7 (1.5-1.9)	4.1 (3.7-4.6)	0.7 (0.5-1.2)	0.8 (0.5-1.4)	1.4 (0.8-2.5)
Chinese	41.7 (36.9-47.2)	3.4 (3.0-3.8)	4.6 (3.4-6.2)	1.6 (1.2-2.2)	0.7 (0.1-5.1)	1.9 (0.3-13.3)
Asian other	23.6 (21.8-25.6)	2.0 (1.8-2.1)	4.4 (3.8-5.1)	1.2 (1.1-1.4)	1.8 (1.0-3.3)	3.3 (1.8-6.1)
Other (eg, Egyptian)	16.4 (15.0-17.9)	1.3 (1.1-1.5)	3.6 (3.1-4.2)	1.2 (1.0-1.4)	0.7 (0.3-1.8)	1.6 (0.6-3.8)

∗ Rate per 100 person-years.

† Model adjusted for sex, Index of Multiple Deprivation, and the number of annual consultations during the study follow-up period.

‡ Compared with Table I, these estimates changed slightly because of a different number of covariates included in the regression model.

**Table E3:** Results after using a more inclusive eczema definition: Incidence rates and rate ratios of eczema by sociodemographic factors stratified by age (N = 675,087 for analysis on sex and Index of Multiple Deprivation and N = 247,798 for analysis on ethnicity)

Characteristic	<1-y-olds (n of eczema = 70,425)		1-4-y-olds (n of eczema = 57,809)		5-17-y-olds (n of eczema = 18,247)	
	Rate∗ (95% CI)	aRR† (95% CI)	Rate∗ (95% CI)	aRR† (95% CI)	Rate∗ (95% CI)	aRR† (95% CI)
Sex						
Male	20.3 (20.1-20.5)	1.3 (1.3-1.3)	5.3 (5.2-5.3)	1.0 (0.9-1.0)	0.9 (0.9-0.9)	0.7 (0.7-0.8)
Female	15.3 (15.1-15.5)	Reference	5.4 (5.4-5.4)	Reference	1.2 (1.2-1.3)	Reference
Index of Multiple Deprivation						
1 (least deprived)	20.0 (19.7-20.3)	1.2 (1.2-1.2)	5.9 (5.8-6.0)	1.1 (1.1-1.2)	1.1 (1.1-1.1)	1.0 (1.0-1.1)
2	17.8 (17.5-18.1)	1.1 (1.1-1.1)	5.4 (5.3-5.5)	1.1 (1.1-1.1)	1.1 (1.0-1.1)	1.0 (1.0-1.1)
3	17.5 (17.2-17.8)	1.1 (1.0-1.1)	5.2 (5.1-5.3)	1.1 (1.0-1.1)	1.1 (1.0-1.1)	1.0 (1.0-1.1)
4	16.9 (16.6-17.2)	1.0 (1.0-1.1)	5.0 (4.9-5.1)	1.0 (1.0-1.1)	1.0 (1.0-1.1)	1.0 (0.9-1.0)
5 (most deprived)	16.5 (16.2-16.8)	Reference	4.9 (4.8-5.0)	Reference	1.1 (1.0-1.1)	Reference
Ethnicity	n of eczema = 32,215		n of eczema = 20,476		n of eczema = 893	
White	16.2 (16.0-16.4)	Reference	5.7 (5.6-5.8)	Reference	1.3 (1.2-1.3)	Reference
Mixed	22.1 (21.0-23.3)	1.4 (1.3-1.5)	6.0 (5.6-6.5)	1.1 (1.0-1.2)	1.5 (1.0-2.2)	1.2 (0.8-1.8)
Black Caribbean	33.6 (30.1-37.5)	2.3 (2.0-2.5)	7.1 (6.0-8.5)	1.3 (1.1-1.6)	2.4 (1.1-5.3)	2.0 (0.9-4.6)
Black African	26.2 (24.6-27.9)	1.8 (1.7-1.9)	5.5 (5.0-6.1)	1.0 (0.9-1.1)	1.8 (1.2-2.8)	1.4 (0.9-2.3)
Black other	33.1 (29.5-37.3)	2.2 (2.0-2.5)	5.7 (4.6-7.0)	1.1 (0.9-1.3)	3.9 (2.2-6.9)	3.5 (2.0-6.3)
Indian	28.8 (27.1-30.5)	1.7 (1.5-1.8)	6.4 (5.9-7.0)	1.1 (1.0-1.2)	1.5 (1.0-2.4)	1.1 (0.7-1.8)
Bangladeshi	34.1 (30.6-38.0)	2.2 (1.9-2.4)	7.8 (6.5-9.2)	1.3 (1.1-1.6)	3.4 (1.7-6.4)	2.3 (1.2-4.5)
Pakistani	23.5 (22.0-25.1)	1.5 (1.4-1.6)	5.9 (5.4-6.5)	1.0 (0.9-1.1)	1.8 (1.2-2.7)	1.3 (0.9-2.0)
Chinese	51.0 (45.5-57.1)	3.1 (2.8-3.5)	7.6 (5.9-9.7)	1.4 (1.1-1.8)	0.8 (0.1-6.0)	0.8 (0.1-5.5)
Asian other	28.0 (26.0-30.2)	1.8 (1.6-1.9)	6.2 (5.5-7.1)	1.1 (1.0-1.2)	2.5 (1.5-4.2)	1.9 (1.1-3.2)
Other	21.2 (19.6-22.9)	1.3 (1.1-1.4)	5.7 (5.1-6.4)	1.0 (0.9-1.2)	1.8 (1.0-3.2)	1.5 (0.8-2.6)

∗ Rate per 100 person-years.

† Model adjusted for sex, Index of Multiple Deprivation, and the number of annual consultations during the study follow-up period.

**Table E4:** Results after using a more strict eczema definition for the first year of life: Incidence rates and rate ratios of eczema by sociodemographic factors stratified by age (N = 675,087 for analysis on sex and Index of Multiple Deprivation and N = 247,798 for analysis on ethnicity)

Characteristic	<1-y-olds (n of eczema = 39,579)		1-4-y-olds (n of eczema = 34,729)		5-17-y-olds (n of eczema = 7,828)	
	Rate∗ (95% CI)	aRR† (95% CI)	Rate∗ (95% CI)	aRR† (95% CI)	Rate∗ (95% CI)	aRR† (95% CI)
Sex						
Male	11.0 (10.9-11.2)	1.3 (1.3-1.3)	2.9 (2.8-2.9)	1.0 (0.9-1.0)	0.4 (0.3-0.4)	0.7 (0.7-0.9)
Female	8.2 (8.1-8.3)	Reference	2.9 (2.9-3.0)	Reference	0.5 (0.5-0.5)	Reference
Index of Multiple Deprivation						
1 (least deprived)	11.2 (11.0-11.4)	1.3 (1.2-1.4)	3.1 (3.1-3.2)	1.2 (1.1-1.2)	0.4 (0.4-0.4)	0.9 (0.9-1.0)
2	9.7 (9.5-9.9)	1.1 (1.1-1.2)	2.9 (2.9-3.0)	1.1 (1.0-1.1)	0.4 (0.4-0.4)	0.9 (0.9-1.0)
3	9.4 (9.2-9.6)	1.0 (0.9-1.1)	2.8 (2.7-2.9)	1.0 (1.0-1.0)	0.4 (0.4-0.4)	0.9 (0.9-1.0)
4	8.9 (8.7-9.1)	1.1 (1.0-1.1)	2.8 (2.7-2.8)	1.0 (1.0-1.1)	0.4 (0.4-0.4)	1.0 (0.9-1.1)
5 (most deprived)	8.7 (8.5-8.9)	Reference	2.7 (2.6-2.8)	Reference	0.4 (0.4-0.5)	Reference
Ethnicity	n of eczema = 18,583		n of eczema = 12,862		n of eczema = 391	
White	8.6 (8.4-8.7)	Reference	3.2 (3.1-3.2)	Reference	0.4 (0.4-0.5)	Reference
Mixed	11.9 (11.2-12.8)	1.4 (1.3-1.5)	3.5 (3.2-3.9)	1.1 (1.0-1.2)	0.5 (0.3-1.0)	1.2 (0.6-2.3)
Black Caribbean	20.6 (18.0-23.6)	2.7 (2.4-3.1)	5.3 (4.3-6.4)	1.8 (1.4-2.2)	1.5 (0.6-3.9)	3.2 (1.2-8.7)
Black African	15.3 (14.1-16.6)	2.0 (1.8-2.2)	3.8 (3.4-4.3)	1.3 (1.1-1.4)	1.1 (0.6-1.8)	2.3 (1.3-4.1)
Black other	18.6 (16.0-21.7)	2.4 (2.1-2.8)	3.8 (3.0-4.9)	1.3 (1.0-1.6)	2.4 (1.2-4.7)	5.2 (2.5-10.5)
Indian	17.3 (16.0-18.6)	1.7 (1.3-2.3)	4.0 (3.6-4.4)	1.3 (1.1-1.4)	0.9 (0.5-1.5)	2.0 (1.2-3.4)
Bangladeshi	23.4 (20.6-26.5)	2.9 (2.6-3.3)	5.1 (4.1-6.2)	1.6 (1.3-2.0)	0.9 (0.3-2.9)	2.0 (0.6-6.1)
Pakistani	14.1 (13.0-15.3)	1.8 (1.6-1.9)	4.0 (3.5-4.4)	1.3 (1.1-1.4)	0.8 (0.4-1.4)	1.7 (0.9-3.0)
Chinese	24.4 (20.9-28.5)	2.8 (2.4-3.3)	4.2 (3.1-5.7)	1.3 (1.0-1.8)	0.7 (0.1-4.6)	1.5 (0.2-10.5)
Asian other	15.1 (13.7-16.7)	1.8 (1.6-2.0)	4.2 (3.6-4.8)	1.3 (1.1-1.5)	1.7 (0.9-3.1)	3.7 (2.0-6.8)
Other	9.9 (8.8-11.0)	1.2 (1.1-1.4)	3.4 (3.0-4.0)	1.1 (1.0-1.3)	0.7 (0.3-1.7)	1.5 (0.6-3.7)

∗ Rate per 100 person-years.

† Model adjusted for sex, Index of Multiple Deprivation, and the number of annual consultations during the study follow-up period.

**Table E5:** Results in the overall child population regardless of the general practitioner registration date related to birth: Incidence rates and rate ratios of eczema by sociodemographic factors stratified by age (N = 1,472,337 for analysis on sex and Index of Multiple Deprivation and N = 392,830 for analysis on ethnicity)

Characteristic	<1-y-olds (n of eczema = 59,840)		1-4-y-olds (n of eczema = 44,111)		5-17-y-olds (n of eczema = 22,527)	
	Rate∗ (95% CI)	aRR† (95% CI)	Rate∗ (95% CI)	aRR† (95% CI)	Rate∗ (95% CI)	aRR† (95% CI)
Sex						
Male	15.9 (15.7-16.1)	1.3 (1.3-1.4)	2.8 (2.8-2.9)	1.0 (1.0-1.0)	0.4 (0.4-0.4)	0.7 (0.7-0.7)
Female	11.7 (11.6-11.8)	Reference	2.9 (2.8-2.9)	Reference	0.5 (0.5-0.5)	Reference
Index of Multiple Deprivation						
1 (least deprived)	15.6 (15.4-15.9)	1.2 (1.2-1.3)	3.1 (3.1-3.2)	1.3 (1.2-1.3)	0.4 (0.4-0.4)	0.9 (0.9-1.0)
2	13.8 (13.5-14.0)	1.1 (1.0-1.1)	2.9 (2.8-2.9)	1.2 (1.1-1.2)	0.4 (0.4-0.4)	0.9 (0.9-0.9)
3	13.6 (13.3-13.8)	1.1 (1.0-1.1)	2.8 (2.7-2.9)	1.1 (1.1-1.2)	0.4 (0.4-0.5)	0.9 (0.9-1.0)
4	13.1 (12.9-13.3)	1.0 (1.0-1.1)	2.7 (2.7-2.8)	1.1 (1.1-1.1)	0.5 (0.5-0.5)	1.0 (1.0-1.0)
5 (most deprived)	12.9 (12.6-13.1)	Reference	2.7 (2.6-2.7)	Reference	0.5 (0.5-0.5)	Reference
Ethnicity	n of eczema = 26,958		n of eczema = 15,269		n of eczema = 1,824	
White	12.4 (12.2-12.5)	Reference	3.1 (3.1-3.2)	Reference	0.5 (0.5-0.5)	Reference
Mixed	17.8 (16.8-18.8)	1.5 (1.4-1.6)	3.6 (3.3-3.9)	1.2 (1.1-1.4)	0.7 (0.5-0.9)	1.4 (1.0-1.9)
Black Caribbean	28.6 (25.5-32.0)	2.5 (2.3-2.8)	5.3 (4.4-6.4)	2.1 (1.7-2.5)	1.7 (1.1-2.5)	3.5 (2.4-5.2)
Black African	21.9 (20.6-23.4)	1.9 (1.8-2.1)	3.9 (3.5-4.3)	1.5 (1.3-1.6)	1.0 (0.8-1.2)	2.1 (1.7-2.6)
Black other	28.5 (25.2-32.2)	2.5 (2.2-2.8)	3.9 (3.2-4.9)	1.4 (1.1-1.8)	1.8 (1.3-2.5)	3.7 (2.6-5.3)
Indian	24.1 (22.7-25.6)	1.9 (1.7-2.0)	4.0 (3.7-4.4)	1.3 (1.2-1.4)	1.0 (0.9-1.3)	2.2 (1.8-2.7)
Bangladeshi	31.2 (28.0-34.8)	2.4 (2.1-2.9)	5.3 (4.4-6.3)	1.6 (1.3-1.9)	1.2 (0.7-2.1)	2.3 (1.3-4.0)
Pakistani	20.1 (18.7-21.5)	1.6 (1.5-1.8)	3.8 (3.5-4.2)	1.1 (0.9-1.3)	1.0 (0.8-1.2)	1.8 (1.5-2.3)
Chinese	41.8 (37.1-41.1)	3.4 (3.0-3.8)	4.6 (3.5-5.9)	1.7 (1.3-2.2)	0.5 (0.2-1.6)	1.1 (0.3-3.3)
Asian other	23.6 (21.8-25.5)	2.0 (1.8-2.1)	4.3 (3.8-4.9)	1.3 (1.2-1.5)	1.2 (0.8-1.6)	2.3 (1.6-3.2)
Other	16.7 (15.4-18.2)	1.3 (1.2-1.5)	3.2 (2.8-3.6)	1.1 (1.0-1.3)	0.9 (0.7-1.2)	1.9 (1.4-2.6)

∗ Rate per 100 person-years.

† Model adjusted for sex, Index of Multiple Deprivation, and the number of annual consultations during the study follow-up period.

## Methods

### Defining eczema

Diagnoses in the CPRD are coded using the Read code system, which is based on a hierarchal clinical classification system and can be cross referenced to the International Classification of Disease.^E8^ We defined a child as having eczema if he or she had 1 diagnostic code for eczema (Read codes M111.00 Atopic dermatitis/eczema, M112.00 Infantile eczema, M113.00 Flexural eczema, M114.00 Allergic intrinsic eczema, M12z100 Eczema NOS) with at least 2 eczema-related treatment codes on separate days within 3 months before or 1 year after the diagnosis. The following eczema-related treatments were used: prescriptions of emollients, topical steroids, topical calcineurin inhibitors (including pimecrolimus and tacrolimus), systemic steroids, systemic immunomodulatory agents (including methotrexate, cyclosporine, azathioprine, and mycophenolate mofetil), and ultraviolet light therapy. Drug prescriptions were identified from the CPRD using drug codes based on the British National Formulary chapters. Ultraviolet light therapy treatments were identified from both the CPRD and Hospital Episode Statistics using Read and OPCS4 codes, respectively.

### Sensitivity analyses

To test the robustness of our results, we conducted 4 sensitivity analyses and compared the results to the main analysis.

1. Missing ethnicity data: Because 18.3% children had missing information on ethnicity, we used multiple imputation to replace missing values for ethnicity by using multinomial logistic regression and created 10 imputed data sets. We combined estimates using Rubin's rule to obtain final estimates and compared the results to the complete case analysis.
2. More inclusive eczema definition: We defined a child as having eczema if he or she had 1 diagnostic code for eczema with at least 1 eczema-related treatment within 3 months before or after the eczema diagnosis.
3. More restrictive eczema definition for the first year of life: To minimize the potential misclassification of eczema diagnosis in the first year of life, we defined a child as having eczema in the first year if he or she had eczema in the first year and prescribed any eczema-related treatment in the second year of life also.
4. Alternative study population: Because we excluded a large proportion of children, that is, those registered with their current primary care physician after the first 3 months of birth (1,097,638 out of 1,838,877; 59.7%), our study population could be less representative of the general pediatric population. Therefore, we re-ran our analysis on the total population identified from the Hospital Episode Statistics–linked CPRD. For this sensitivity analysis, to ensure we included only incident diagnoses and not prevalent ones in children registered after the first year of life, we excluded the first 12 months after the current registration date and children whose first eczema diagnosis was within this first 12 months period were also excluded.
